# Duodenal and colonic mucosal S100A8/A9 (calprotectin) expression is increased and correlates with the severity of select histologic lesions in dogs with chronic inflammatory enteropathy

**DOI:** 10.1186/s12917-024-04256-9

**Published:** 2024-09-06

**Authors:** Jasmin Nestler, Pernilla Syrjä, Susanne Kilpinen, Clara Antunes Moniz, Thomas Spillmann, Mohsen Hanifeh, Romy M. Heilmann

**Affiliations:** 1 Department for Small Animals, College of Veterinary Medicine, University of Leipzig, Leipzig, SN Germany; 2 Department of Veterinary Biosciences, Faculty of Veterinary Medicine, University of Helsinki, Helsinki, Finland; 3 Department of Equine and Small Animal Medicine, Faculty of Veterinary Medicine, University of Helsinki, Helsinki, Finland; 4 Bühlmann Laboratories, Schönenbuch, Switzerland

**Keywords:** Calgranulin, Canine, Chronic enteropathy, Inflammatory bowel disease, Mucosa extract

## Abstract

**Background:**

Calprotectin, a damage-associated molecular pattern protein of the S100/calgranulin family, is a potential marker of gastrointestinal inflammation in dogs and mainly originates from activated macrophages and granulocytes. Increased calprotectin concentrations are reported in feces and serum samples from dogs with chronic inflammatory enteropathy (CIE), but mucosal calprotectin expression has not been extensively investigated in canine CIE. Thus, we aimed to evaluate gastrointestinal mucosal concentrations of calprotectin in 62 dogs (44 dogs with CIE compared to 18 healthy Beagles) using a particle-enhanced turbidimetric immunoassay method. Additionally, we assessed the relationship of gastric, duodenal, jejunal, ileal, and colonic mucosal calprotectin levels with the clinical disease severity (canine clinical inflammatory bowel disease activity index, CIBDAI), histopathologic findings, clinical outcome, and serum albumin concentrations to further evaluate the potential of calprotectin as a biomarker for CIE.

**Results:**

Mucosal calprotectin concentrations in dogs with CIE were significantly higher in the duodenum (median: 276.2 μg/g) and colon (median: 298.2 μg/g) compared to healthy controls (median: 94.3 μg/g, *P* = 0.0039; and median: 112.0 μg/g, *P* = 0.0061). Similar numerical differences in the ileum and cecum were not statistically significant, and mucosal calprotectin concentrations correlated significantly among the different gastrointestinal segments. Histologic lesion severity was linked to mucosal calprotectin concentrations for inflammatory and structural histology criteria in the duodenum and colon (all *P* < 0.05). Higher mucosal calprotectin levels in the duodenum and across all segments correlated with lower serum albumin concentrations (both *P* < 0.05); duodenal mucosal calprotectin concentrations were more than sixfold higher in hypoalbuminemic dogs (median: 1441 µg/g, *n* = 4) than normoalbuminemic dogs (median: 227 µg/g, *n* = 40). There was no significant association of mucosal calprotectin levels with CIBDAI scores or individual clinical outcomes.

**Conclusions:**

These results show that duodenal and colonic mucosal calprotectin concentrations are increased in dogs with CIE, providing further supporting evidence for the diagnostic potential of fecal calprotectin (presumably reflecting mucosal) concentrations and in dogs with CIE. Further longitudinal research is needed to assess changes in mucosal calprotectin concentrations with clinical response to treatment vs. mucosal disease remission and to determine the clinical utility of fecal calprotectin concentrations to diagnose and monitor dogs with CIE in clinical practice.

**Supplementary Information:**

The online version contains supplementary material available at 10.1186/s12917-024-04256-9.

## Introduction

Chronic inflammatory enteropathies (CIE) describe a disease complex with heterogeneous clinical signs that requires infectious (e.g., parasitic), neoplastic, and mechanical gastrointestinal conditions and extra-intestinal (e.g., hepatic or pancreatic) etiologies to be ruled out [1–3]. Great strides to better understand this disease complex have been made over the past two decades but the pathogenesis remains incompletely understood [1]. Based on the current consensus, CIE is characterized by an exaggerated immune response, including elements of both innate and adaptive immunity [1]. They are classified as food-responsive enteropathy (FRE), antibiotic-responsive enteropathy (ARE, now proposed to be named "idiopathic intestinal dysbiosis"), steroid-responsive enteropathy (SRE), and non-responsive enteropathy (NRE) [1]. Protein-losing enteropathy can occur as a severe form of CIE due to secondary intestinal lymphangiectasia (ILE) resulting from marked inflammation, but can also result from primary ILE [1–3]. Because dogs with chronic gastrointestinal signs present a large proportion of internal medicine patients in veterinary practice [4] and are commonly subjected to sequential diagnostic investigation for a possible CIE [1], non-invasive inflammatory biomarkers such as fecal calprotectin have become an attractive focus of clinical research [5]. However, more studies are needed to fully characterize the role of these molecules in the pathogenesis of CIE and determine their clinical utility in dogs with chronic signs of gastrointestinal disease.

Calprotectin, the S100A8/A9 protein complex, belongs to the group of damage-associated molecular pattern (DAMP) molecules and is a ligand for the innate immune receptor Toll-like receptor (TLR)-4, which plays a role in acute and chronic inflammation [6]. Predominantly activated neutrophils, infiltrating macrophages (MФ), and – under certain conditions such as chronic inflammation or malignant transformation – also epithelial cells are described to express S100A8/A9 [6–9]. Recently, single-cell ribonucleic acid (RNA) sequencing of the duodenal mucosal cell populations revealed an overexpression of the S100A8 subunit of calprotectin also localized to mucosal T cells in canine CIE [9]. However, the functional implication of the lack of the S100A9 subunit in these cells requires further study.

Another recent investigation into gastrointestinal mucosal calprotectin expression in dogs with CIE compared to healthy controls identified calprotectin-positive cells in all gastrointestinal segments evaluated (stomach, duodenum, ileum, and colon) and with a predominant localization to the lamina propria [10]. Numbers of lamina propria calprotectin-positive cell counts were significantly lower in the gastric mucosa compared to the duodenal, ileal, and colonic mucosa in this study, but no differences were seen in calprotectin positivity among the intestinal segments [10].

Ambiguous results have been reported for the relationship between the severity of MФ/neutrophilic cellular infiltrates in the intestinal mucosa and calprotectin-positive cell counts [10] or fecal calprotectin concentrations [11] in dogs. Whereas calprotectin-positive cells in the lamina propria correlated positively with the extent of neutrophil and MФ infiltration in the ileal mucosa, there was no significant correlation between the numbers of calprotectin-positive cells and the severity of MΦ or neutrophil infiltration in any other gastrointestinal segments [10]. Fecal calprotectin concentrations in dogs with CIE were significantly linked to the severity of histologic inflammatory lesions, particularly the lymphocytic infiltration of the lamina propria, but did not reflect the numbers of neutrophils and MΦ in the intestinal lamina propria [11]. Particularly the latter was an unexpected finding given that calprotectin is predominantly expressed by activated neutrophils and MΦ and was shown to correlate with the number of infiltrating neutrophils in human patients with chronic intestinal inflammation associated with inflammatory bowel disease (IBD) [12]. This raised the question of whether not merely the number of calprotectin-expressing cells but rather the ability or level of these cells to express calprotectin is evaluated when measuring calprotectin concentrations in fecal samples and/or whether other lamina propria cells in the canine gastrointestinal mucosa might also express calprotectin [5, 10]. To evaluate this hypothesis, gastrointestinal mucosal levels of calprotectin expression remain to be further studied in dogs with CIE. Thus, this study aimed to evaluate calprotectin concentrations in extracts of mucosal biopsies obtained from different segments of the gastrointestinal tract from dogs with CIE (1) in comparison to mucosal calprotectin concentrations in healthy control dogs and (2) in relation to clinical, clinicopathologic, histologic and outcome measures in dogs diagnosed with CIE.

## Results

### Study population

**Fig. 1:**
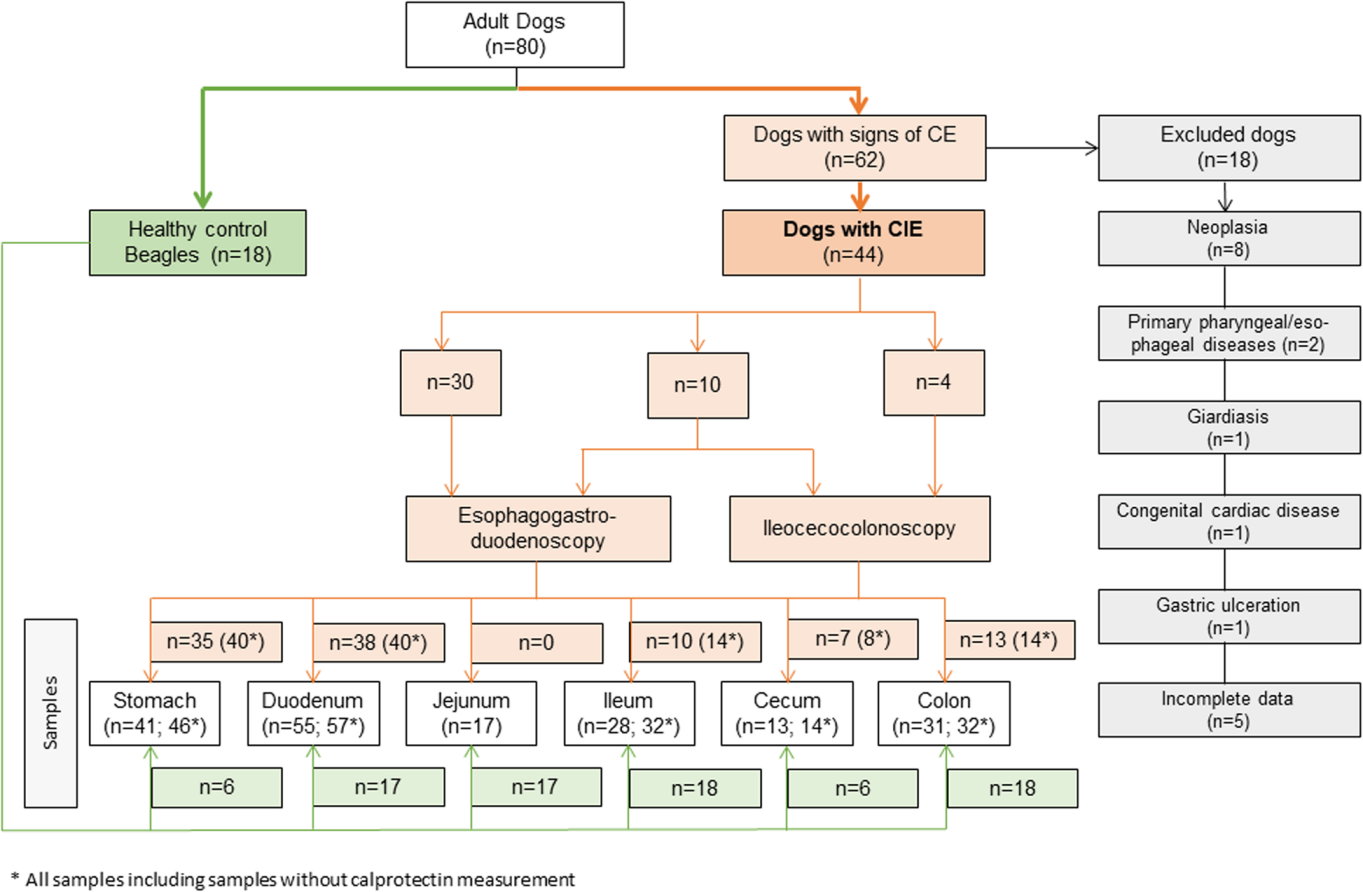
Study flowchart summarizing the dogs enrolled in the study

Of the 62 dogs considered for inclusion in the study, 18 dogs with chronic gastrointestinal signs were excluded from further analysis. Eight of these dogs were diagnosed with gastrointestinal neoplasia (4 gastric carcinomas, 2 small intestinal lymphomas, 1 undifferentiated large intestinal round cell tumor, and 1 rectal adenocarcinoma). Two dogs had primary pharyngeal or esophageal diseases, and one dog each was positive for *Giardia* on a fecal examination, had congenital cardiac disease undergoing surgery, or had an acute worsening of clinical signs with gastric ulceration. Five dogs had to be excluded due to incomplete data. Hence, 44 dogs with CIE were included in the further analyses (Fig. 1), and 18 healthy Beagle dogs served as a control group. Medium age was 5.3 years (range: 1–13 years) with 19 females (14 spayed, 5 intact) and 25 male dogs (12 neutered, 13 intact) in the CIE group and was 9.5 years (range: 6–13 years) with 10 females (3 spayed, 7 intact) and 8 males (5 neutered, 3 intact) in the healthy control group.

Thirty of the 44 dogs with CIE underwent esophagogastroduodenoscopy, 10 dogs had both esophagogastroduodenoscopy and ileocecocolonoscopy, and ileocecocolonoscopy alone was performed in 4 dogs. In addition to 116 diagnostic biopsies, a total of 103 mucosal biopsies were collected by endoscopy from 5 different segments of the gastrointestinal tract of the dogs with CIE, including the stomach (*n* = 35), duodenum (*n* = 38), ileum (*n* = 10), colon (*n* = 13), and cecum (*n* = 7). From the healthy control group, a total of 82 mucosal biopsies were obtained for diagnostic purposes, and additional 65 biopsies were obtained from the stomach (*n* = 6), duodenum and jejunum (each *n* = 17), ileum (*n* = 18), colon (*n* = 18), and cecum (*n* = 6). The clinical and clinicopathological characteristics of both groups of dogs are summarized in Table 1. Based on the histological examination of gastrointestinal samples from healthy Beagles, the median total histology score in all tissue specimens was 0 (range 0–4), classifying all findings in healthy dogs as insignificant.

**Table 1:** Patient characteristics, clinical findings, and clinicopathologic parameters in dogs with chronic inflammatory enteropathies (CIE, *n* = 44) and healthy controls (*n* = 18) included in the study

Characteristic	CIE group	Control group	*P*-value
Age in years, median (IQR)	**5.0 (2.5–8.0)**	**10.5 (6.0–11.0)**	** < 0.0001**
Sex, male (neutered) / female (spayed)	26 (12) / 18 (13)	8 (5) / 10 (3)	0.2934
Body weight in kg, median (IQR)	17.4 (8.0–28.6)^a^	15.2 (12.5–18.4)^b^	0.4480
Breed, n (%)			
- pure-bred	39 (89%)	18 (100%)	0.0576
- mixed breed	5 (11%)	0 (0%)	
Number of gastrointestinal sites biopsied, median (IQR)	2 (2–3)	4 (4–6)	–
Histologic lesion score, median (IQR)	2 (1–2)	0.5 (0–0.5)	** < 0.0001**
Max. severity of histologic lesions, n (%)			
- none/minimal (histologic score = 0)	0	**2 (11%)**	
- mild (histologic score = 1)	**10 (23%)**	**16 (89%)**	** < 0.0001**
- moderate (histologic score = 2)	**27 (61%)**	0	
- severe (histologic score = 3)	**7 (16%)**	0	
***Clinical parameters***			
CIBDAI score^c^, median (IQR)	5 (3–7)	–	–
Clinical disease severity^c^, n (%)		–	–
- insignificant (CIBDAI score ≤ 3)	9 (26.5%)		
- mild (CIBDAI score 4–5)	12 (35%)		
- moderate (CIBDAI score 6–8)	9 (26.5%)		
- severe (CIBDAI score ≥ 9)	4 (12%)		
***Clinicopathologic parameters***			
Serum albumin in g/L, median (IQR)	31.5 (29.0–34.7)^d^	32.5 (31.1–33.4)	0.4602
Hypoalbuminemia, n (%)	4 (10%)^d^	0 (0%)	0.0748

*CIBDAI* canine inflammatory bowel disease activity index, *IQR* Interquartile range

^a^available from *n* = 43 dogs; ^b^available from *n* = 16 dogs; ^c^available from* n* = 34 dogs; ^d^available from *n* = 39 dogs

There were no significant differences between both groups except for the healthy control group being significantly older than the group of dogs with CIE and the healthy group being solely Beagle dogs. Breeds of dogs with CIE included the following: Collie and Shetland Sheepdog (each *n* = 3), German Shepherd dog, Poodle, Rottweiler, and Staffordshire Bull Terrier (each *n* = 2); Alaskan Malamute, Bichon Frise, Border Terrier, Chihuahua, Chow Chow, Dachshund, Dalmatian, English Bulldog, Golden Retriever, Havanese, Irish Terrier, Jack Russell Terrier, Labrador Retriever, Mudi, Norwegian Lundehund, Parson Russell Terrier, Rhodesian Ridgeback, Siberian Husky, Silky Terrier, Spanish Water Dog, St. Bernard, Toy Poodle, Welsh Corgi, West Highland White Terrier, and White Shepherd dog (each *n* = 1); and mixed breed dog (*n* = 5).

The canine inflammatory bowel disease activity index (CIBDAI) scores indicated mild to moderate clinical disease severity in the CIE group of dogs. The most common clinical sign was diarrhea with changes in stool consistency in 25 dogs (74%; severity scores ranging from 1–3, median: 2) and increased frequency of defection in 21 dogs (62%; severity scores: 1–3, median: 1), followed by vomiting (*n* = 24, 71%; severity scores: 1–3, median: 1), hypo- or anorexia (*n* = 19, 56%; severity scores: 1–2, median: 1), weight loss (*n* = 14, 41%; severity scores: 1–3, median: 1), and attitude and/or activity changes (*n* = 10, 29%; severity scores: 1–2, median: 1.5). Numbers of sections biopsied ranged from 6–16 (median: 8) for the stomach, from 2–11 (median: 8) for the duodenum, from 1–8 (median: 7) for the ileum, and from 6–10 (median: 8) for the colon; these numbers were not documented for the jejunum and cecum.

Moderate to marked hypoalbuminemia was detected in 4 dogs (serum albumin concentration 11–13 g/l, median: 12.2 g/l). Clinical outcomes were available from 34 dogs, of which, based on the response after an elimination diet trial or antibiotic or corticosteroid treatment, 10 (29%) were classified as FRE, 6 dogs (18%) as ARE, 13 dogs (38%) were diagnosed as IRE, and 5 dogs (15%) as NRE. Non-responsive dogs (NRE) were euthanized because of severe clinical signs and unfavorable responses to treatment. Of the dogs with hypoalbuminemia, 3 dogs were classified as SRE and 1 dog as NRE.

### Mucosal S100A8/A9 (calprotectin) concentrations

**Fig. 2:**
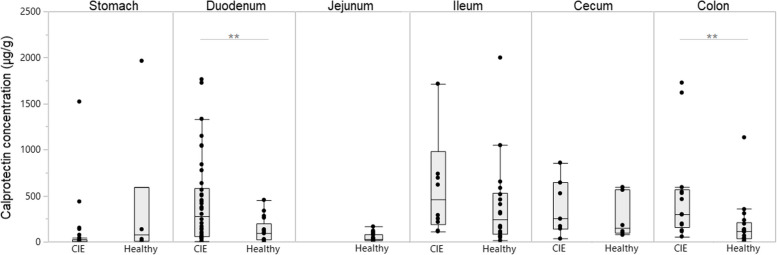
S100A8/A9 (calprotectin) concentrations in gastrointestinal mucosal extracts from dogs with CIE and healthy controls. Significantly higher calprotectin concentrations were measured in mucosal extracts from the duodenum and colon in dogs with CIE compared to healthy Beagles (*P* = 0.0039 and *P* = 0.0061), whereas higher calprotectin concentrations in ileal and cecal mucosal extracts from CIE dogs did not reach significant difference from controls. ** indicates *P* < 0.01

Dogs with CIE had significantly higher calprotectin concentrations in mucosal extracts of the duodenum (median: 276.2 µg/g, interquartile range [IQR]: 61.2–584.7 µg/g) and colon (median: 298.2 µg/g, IQR: 155.3–566.9 µg/g) than healthy Beagle dogs (median: 94.3 µg/g, IQR: 23.6–200.9 µg/g, *P* = 0.0039; and median: 112.0 µg/g, IQR: 34.7–205.6 µg/g, *P* = 0.0061; Fig. 2). Dogs with CIE also had higher mucosal calprotectin concentrations than healthy Beagle dogs in the ileum (median: 457.4 µg/g, IQR: 194.8–985.2 µg/g *vs.* median: 242.7 µg/g, IQR: 88.3–535.4 µg/g; *P* = 0.1436) and cecum (median: 249.7 µg/g, IQR: 142.8–641.8 µg/g *vs.* median: 147.2 µg/g, IQR: 92.7–570.0 µg/g; *P* = 0.1436), but these differences did not reach statistical significance. Calprotectin concentrations in mucosal extracts of the stomach did not differ between dogs with CIE (median: 9.0 µg/g, IQR: 1.8–28.5 µg/g) and healthy Beagles (median: 81.6 µg/g, IQR: 7.1–593.8 µg/g; *P* = 0.1166) but were available from only *n* = 6 healthy dogs; jejunal mucosal calprotectin concentrations were measured in only the healthy Beagle group (median: 25.7 µg/g, IQR: 13.2–81.2 µg/g).

Mucosal calprotectin concentrations were significantly correlated between the duodenum and colon (*ρ* = 0.55, *P*_*corr*_ = 0.0180), ileum and jejunum (*ρ* = 0.78, *P*_*corr*_ = 0.0010), and between ileum and colon (*ρ* = 0.65, *P*_*corr*_ = 0.0010). In dogs with CIE, a significant correlation of mucosal calprotectin concentrations in the stomach with those in the duodenum remained (*ρ* = 0.47, *P*_*corr*_ = 0.0315), whereas jejunal and ileal mucosal calprotectin concentrations were significantly correlated in healthy dogs (*ρ* = 0.78, *P*_*corr*_ = 0.0010) (Suppl. Table 1).

Eight dogs were excluded due to a diagnosis of gastrointestinal neoplasia. Of the four dogs with gastric carcinoma, gastric mucosal calprotectin concentrations were determined in three dogs (21, 57, and 150 μg/g), overlapping those detected in healthy controls and dogs with CIE. In contrast, the two dogs diagnosed with duodenal lymphoma had mucosal calprotectin levels in the duodenum that were higher than in healthy controls but overlapped with the CIE group (423 and 1,445 μg/g). One dog with an undifferentiated large intestinal round cell tumor had a colonic mucosal calprotectin level of 34,312 μg/g (mucosal extract noted to have a hemolytic appearance), and another dog with a rectal adenocarcinoma of 1,979 μg/g in the rectum mucosa.

### Association of mucosal S100A8/A9 concentrations with clinical variables

In dogs with CIE, we did not observe any significant association between the clinical disease activity prior to treatment (assessed by CIBDAI score) and mucosal calprotectin concentrations in extracts of the different segments or their cumulative or average calprotectin concentrations (all *P* > 0.05). Of the individual CIBDAI parameters, only the severity of vomiting was moderately inversely correlated with the cumulative (*ρ* = -0.45, *P*_*corr*_ = 0.0474) and average calprotectin concentrations (*ρ* = -0.49, *P*_*corr*_ = 0.0204) across all gastrointestinal segments.

Considering all dogs, serum albumin concentration was moderately inversely correlated with the mucosal calprotectin concentration in the duodenum (*ρ* = -0.39, *P*_*corr*_ = 0.0264) and across all segments (*ρ* = -0.33, *P*_*corr*_ = 0.0260). Hypoalbuminemic dogs with CIE had higher duodenal mucosal calprotectin levels (median: 1,440.9 µg/g, IQR: 1,071.2–1,757.2 µg/g) than normoalbuminemic dogs (median: 206.0 µg/g, IQR: 62.8–506.2 µg/g), but the statistical comparison was limited given the small number of hypoalbuminemic dogs (*n* = 4) in the study.

More severe select morphologic and/or inflammatory lesions and/or higher cumulative lesion scores in the duodenum (*P* = 0.0169) and colon (*P* = 0.0348) were seen in dogs with IRE or NRE compared to FRE or ARE dogs. The severity of several individual and cumulative segmental histologic lesions was moderate to strongly correlated with the concentration of calprotectin in mucosal extracts of the duodenum and colon (Table 2, Suppl. Table 1), whereas a moderate correlation with only the lymphoplasmacytic infiltration was seen for the stomach and cecum (Table 2). Neutrophils within the inflammatory infiltrate were only detected in dogs with CIE and were associated with significantly higher mucosal calprotectin concentrations in the duodenum (median: 455.1 µg/g, IQR: 177.1–1,100.9 µg/g *vs.* median: 105.5 µg/g, IQR: 50.8–374.0 µg/g; *P* = 0.0220) and a similar but statistically not significant trend was seen for the colon (median: 592.0 µg/g, IQR: 528.6–1,618.3 µg/g *vs.* median: 245.6 µg/g, IQR: 121.1–482.1 µg/g; *P* = 0.0759).

**Table 2:**
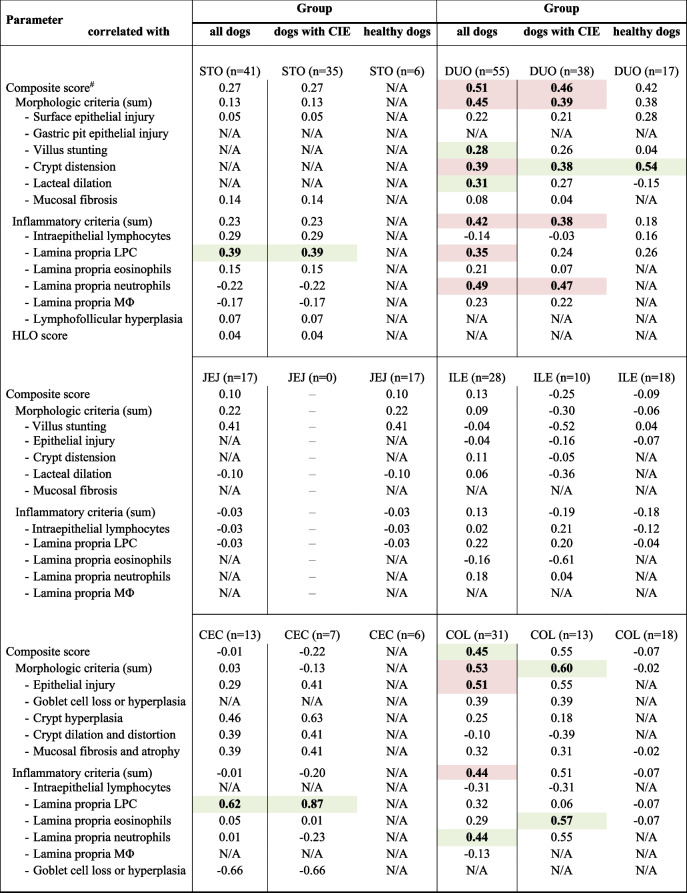
Correlation of mucosal calprotectin concentrations with histologic findings. Shown are the correlations among calprotectin concentrations in mucosal extracts and the severity of morphologic and inflammatory histologic lesions in the stomach, duodenum, jejunum, ileum, cecum, and colon in dogs with CIE (*n*=44) and healthy controls (*n*=18)

*HLO* *Helicobacter*-like organisms, *LPC* lymphocytes/plasma cells, *ΜΦ* macrophages, *N/A* not applicable

Cell counts highlighted in red indicate a statistically significant correlation (*P*_corr_<0.05), and those cells highlighted in green indicate a trend for a significant correlation (*P*<0.05, but *P*_corr_>0.05)

^#^lesions in the stomach evaluated for fundus and antrum (combined)

CIBDAI scores prior to treatment were highest in dogs with NRE but were not significantly different among the groups of dogs with CIE classified based on treatment response (Table 3). CIBDAI scores were not correlated with the severity of gastrointestinal histologic lesions nor with mucosal calprotectin concentration in any of the gastrointestinal segments evaluated or along the gastrointestinal tract (all *P* > 0.05).

**Table 3:** Association of mucosal calprotectin concentrations and clinical disease activity scores with disease subclassification in dogs with CIE (*n* = 34). Shown are the calprotectin concentrations in gastrointestinal mucosal extracts from dogs with CIE responding to different sequentially implemented treatments

Gastrointestinal segment	Calprotectin concentration in µg/g, median (IQR)								*P*
	**FRE**		**ARE**		**IRE**		**NRE**		
Stomach	*n* = 9	15.6 (5.4–35.8)	*n *= 5	7.8 (0.3–18.0)	*n* = 11	6.7 (4.0–20.7)	*n* = 5	2.5 (0–146.3)	0.5674
Duodenum	*n* = 10	313.0 (97.6–635.8)	*n* = 5	90.8 (50.8–253.6)	*n* = 12	379.8 (81.4–1,059.1)	*n* = 3	1,044.1 (50.0–1,336.9)	0.3872
Ileum	*n* = 2	1,170.3 (622.6–1,718.0)	*n* = 3	741.0 (254.7–2,593.8)	*n* = 3	292.1 (115.4–697.7)	*n* = 0	–	0.4054
Colon	*n* = 4	560.3 (355.8–1,361.7)	*n* = 4	191.5 (139.6–455.4)	*n* = 2	1,010.8 (294.9–1,726.7)	*n* = 0	–	0.1587
All segments (avg)	* n* = 10	153.8 (75.8–270.8)	*n* = 6	34.5 (24.2–223.1)	*n* = 13	128.2 (26.1–371.1)	*n* = 5	13.1 (0–334.2)	0.5396
All segments (sum)	*n* = 10	449.0 (161.9–633.0)	*n* = 6	101.8 (63.8–568.0)	*n* = 13	256.4 (70.2–729.5)	*n* = 5	26.3 (0–668.4)	0.5309
**Scoring system**	**CIBDAI score, median (IQR)**								***P***
	**FRE**		**ARE**		**IRE**		**NRE**		
CIBDAI score	*n* = 9	4 (3–7.5)	*n* = 4	5.5 (2–6)	*n* = 13	4 (2–6)	*n* = 3	7 (4–9)	0.4874

*ARE* Antibiotic-responsive enteropathy, *avg* average, *CIBDAI* Canine inflammatory bowel disease activity index score, *CIE* Chronic inflammatory enteropathy, *FRE* Food-responsive enteropathy, *IQR* Interquartile range, *IRE* Immunosuppressant-responsive enteropathy, *NRE* Non-responsive enteropathy

## Discussion

This study further evaluated the gastrointestinal mucosal expression of canine calprotectin, the S100A8/A9 protein complex, and compared calprotectin concentrations in the supernatants of mucosa extracts from dogs with CIE to those from healthy Beagles and to the severity of clinical signs, clinicopathologic findings, histologic lesions, and clinical outcomes.

Compared to healthy Beagle dogs, dogs with CIE had significantly higher calprotectin concentrations in mucosal extracts obtained from the duodenum and colon. This finding is consistent with a previous study investigating the number of calprotectin-expressing cells but not evaluating the protein concentrations within the gastrointestinal mucosa [10]. In this last study, ileal lamina propria calprotectin-positivity was directly correlated with the extent of segmental neutrophil and MФ infiltration in the ileum of dogs with CIE. However, a similar relationship was not seen in any other gastrointestinal segment evaluated, and an inverse correlation was detected between lamina propria S100A12-positivity (another inflammatory protein in the same subfamily) and the severity of MФ infiltration in the duodenum [10]. Another study evaluating mucosal extracts [13] documented significantly higher duodenal mucosal S100A12 concentrations if the inflammatory infiltrate contained predominantly neutrophils and macrophages.

These different findings would support our hypothesis that calprotectin concentrations expressed in and released from the gastrointestinal mucosa do not necessarily reflect the number of infiltrating calprotectin-expressing cells but rather their capacity to express calprotectin and, thus, their inflammatory activity [10, 11, 14] and might point to a role of calprotectin in the pathogenesis of canine CIE.

Dogs with CIE had higher mucosal calprotectin concentrations in the ileum and cecum than healthy Beagle dogs, but these differences did not reach statistical significance. This direction of change is in line with a positive correlation between S100A8/A9-positive cells with the severity of MΦ and neutrophil infiltration in the ileum of dogs with CIE [10] and could be explained by calprotectin being expressed by predominantly macrophages and neutrophils [9]. Many dogs with CIE develop hypocobalaminemia [15–17], previously believed to result from cobalamin uptake being regulated in the ileum and reflecting ileal disease severity, although this theory was recently challenged [15]. In addition to hypocobalaminemia, ileal lesion severity is also reflected by hypoalbuminemia and intestinal lymphangiectasia, particularly in the ileum, has been linked to hypoalbuminemia (serum albumin concentration < 25 g/L) [18, 19]. An inflated type II statistical error might explain the lack of statistical significance for the difference in ileal and cecal mucosal calprotectin concentrations between groups of dogs in our study.

Gastric mucosal calprotectin concentrations did not differ between dogs with CIE and healthy Beagles. This result agrees with a recent immunohistochemistry-based study documenting significantly lower numbers of lamina propria calprotectin-positive cell counts in the stomach compared to the duodenum, ileum, and colon [10]. It would also support the conclusion that the stomach is infrequently involved in the mucosal inflammatory process in dogs with CIE [20] and is not a major contributor to the intraluminal release of calprotectin, accounting for the differences in its fecal concentrations between dogs with CIE and healthy controls.

In dogs with CIE, we did not observe a significant association between the clinical disease activity (as assessed by the CIBDAI score) prior to initiation of treatment and mucosal calprotectin concentrations in extracts of tissue samples from the different segments or the cumulative or average calprotectin concentrations across all segments. This agrees with the previous immunohistochemistry study showing mucosal calprotectin-positivity is not associated with the extended canine chronic enteropathy clinical activity index (CCECAI) score [10]. This suggests that the expression of innate immune signals is not necessarily reflected by the patient’s clinical signs and might fit with the consideration of clinical *vs.* mucosal disease remission as important clinical endpoints [1].

In our study, CIBDAI scores were not correlated with the severity of gastrointestinal histologic lesions. The same results were reported by others [17], whereas another study documented a significant correlation of CCECAI or CIBDAI scores with cumulative histologic scores for the duodenum and colon [20]. However, of the individual CIBDAI parameters, we found more severe vomiting linked to lower cumulative and mean calprotectin concentrations across all gastrointestinal segments. With vomiting being a clinical sign of upper gastrointestinal disease, this would align with the overall lower mucosal calprotectin levels in the stomach. Interestingly, serum calprotectin (and S100A12) concentrations were also significantly lower in a study of Miniature schnauzers with pancreatitis and vomiting [21]. Thus, the stomach may not be substantially involved in the expression of calprotectin, and a contribution of extraintestinal causes such as pancreatitis to vomiting in dogs with CIE cannot be excluded.

Hypoalbuminemic dogs with CIE had higher duodenal mucosal calprotectin levels than normoalbuminemic dogs, but the statistical comparison was limited given the small number of hypoalbuminemic dogs in the study, and the interpretation of the group comparisons was unchanged when hypoalbuminemic CIE dogs were excluded from the analysis. Similarly, higher intraepithelial calprotectin-positive cell counts in the duodenum, presumably participating in the first line of epithelial defense, were previously observed in hypoalbuminemic dogs than in normoalbuminemic patients [10]. This aligns with marked endoscopic lesions in the duodenum and severe CIE, with secondary protein-losing enteropathy (serum albumin concentrations < 20 g/L) being predictive of more severe disease and negative outcomes [17]. However, beyond simply reflecting more severe inflammation and particularly higher lamina propria neutrophil counts in hypoalbuminemic dogs with CIE [22], hypoalbuminemia and increased mucosal calprotectin levels may also result from intraluminal leakage caused by primary intestinal lymphangiectasia. As previously reported [17], more severe histologic lesions in the duodenum and colon were seen in dogs with IRE or NRE than in FRE or ARE dogs. Contrasting our findings, dogs with IRE or NRE had significantly higher fecal calprotectin concentrations than dogs with FRE or ARE [11], and a similar tendency was detected in cats with CIE [23].

The severity of several individual and cumulative segmental histologic lesions was moderate to strongly correlated with the concentration of calprotectin in mucosal extracts of the duodenum and colon, whereas a moderate correlation with only the lymphoplasmacytic infiltration was seen for the stomach and cecum. Dogs with CIE mostly have a predominating lymphoplasmacytic mucosal cellular infiltrate [24–26]. However, the role of mucosal lymphocytes and plasma cells in canine CIE is unclear as their numbers do not correlate with clinical disease scores, inflammatory markers such as serum C-reactive protein (CRP), or patient outcomes [17, 27–30]. Macrophages are a group of inflammatory cells involved in the expression of calprotectin and can be classified as M1 (pro-inflammatory), M2 (anti-inflammatory), and other “in-between” phenotypes [31]. Calprotectin is particularly a marker of early differentiated (M1) macrophages, whereas a different antigen signature accounts for duodenal resident (M2) macrophages [32–34]. Higher mucosal calprotectin concentrations might, therefore, reflect higher histologic lesion severity in some segments of the gastrointestinal tract. However, we propose that the activity of calprotectin-expressing cells rather than only the number of these cells is relevant. This would also explain why mucosal calprotectin concentrations and calprotectin-positive cell counts [10] are not or only partially correlated with the severity of microscopic lesions overall and the inflammatory infiltrate. Neutrophils within the inflammatory infiltrate were detected in some dogs with CIE and were associated with significantly higher mucosal calprotectin concentrations in the duodenum, with a similar trend seen for the colon. This is consistent with calprotectin secretion by neutrophils in addition to activated macrophages [6].

Eight dogs diagnosed with different types of gastrointestinal neoplasia had to be excluded from the analysis as this subgroup was too small and heterogeneous for further statistical comparison with the CIE and/or healthy control group of dogs. Dogs with gastric carcinoma had gastric mucosal calprotectin concentrations overlapping those detected in healthy controls and dogs with CIE. This leads us to conclude that the gastric mucosa has little involvement in calprotectin expression in CIE and neoplasia. In line with this, fecal calprotectin is not a useful diagnostic biomarker to distinguish between gastric cancer and health in human patients [35]. Different observations were made with duodenal calprotectin levels in dogs diagnosed with duodenal lymphoma. Those levels in the duodenum were higher than in healthy controls but overlapped with the CIE group, making it less likely for this biomarker to differentiate dogs with CIE from those with small intestinal lymphoma, as has recently been shown in cats [23]. One dog with a rectal adenocarcinoma had a very high calprotectin level in the rectum mucosa. Significant overexpression of mucosal calprotectin in colorectal adenomas and carcinomas has been described in humans [36], and fecal calprotectin may aid in discriminating colorectal cancer from non-malignant gastrointestinal conditions in humans [35]. Whether calprotectin is expressed by the malignant epithelial or round cell population or primarily localized to cells of the tumor-associated inflammation as shown with canine bladder and prostatic cancer [37, 38] remains to be determined. Thus, studies involving larger cohorts of dogs affected with gastrointestinal cancer are needed to evaluate further the role of calprotectin in gastrointestinal dysplasia and malignant transformation.

We acknowledge the study's main limitations, including only dogs of a single breed (Beagle) as the control group and this group being older than the dogs in the CIE group. However, older dogs would be expected to be more likely to have histologic lesions in the gastrointestinal tract and potentially more susceptible to certain diseases, adding rigor to the evaluation performed. Still, these results warrant confirmation by further studies. Possible misclassification of CIE based on the response to treatment, especially refuting a diagnosis of FRE based on adequate and sequential dietary trials, remains another limitation. Further, the differences in lifestyles between the groups of dogs, with healthy controls being Beagle dogs that lived in a colony and dogs with CIE being pet dogs living in the home environment of the owner, might present a potential confounding factor. The housing of these dogs as a group (as mandated by animal welfare policies) also excluded the possibility of reliable fecal scoring. Another limitation is the different mucosal tissue biopsy sampling methods in dogs with CIE (undergoing endoscopic biopsy) vs. healthy controls (post-mortem mucosal sample collection at necropsy). Also, spatial differences between the biopsies obtained for histopathologic evaluation and those obtained to be extracted for calprotectin analysis may account for some variation. Pathologist bias in the histopathologic evaluation of mucosal tissue biopsies can also not be entirely excluded. Further, frozen storage of the mucosal tissue samples until further examination might have affected calprotectin concentrations. However, studies showed remarkable stability of calprotectin concentrations in fecal samples that were stored frozen [39–41], and it is reasonable to assume similar stability of calprotectin in frozen mucosal samples.

The results of this study provide further support for an increased intestinal mucosal calprotectin expression in dogs with CIE and potentially other chronic gastrointestinal conditions (i.e., cancer). It is suspected but remains to be proven that this increased mucosal calprotectin expression contributes to increased fecal calprotectin concentrations in dogs with CIE. Further prospective research is needed to assess the value of determining mucosal calprotectin concentrations in clinical practice, the relationship between mucosal calprotectin levels and fecal calprotectin concentrations with several gastrointestinal diseases, and its correlation with other clinicopathologic (e.g., inflammatory) and/or other biomarkers in dogs with CIE.

## Methods

### Study population

Considered for inclusion in the study were dogs with chronic gastrointestinal signs (n = 62) that presented to the Small Animal Internal Medicine service at the Department of Equine and Small Animal Medicine, Faculty of Veterinary Medicine, University of Helsinki, Finland, between February 2013–July 2016 for further diagnostic evaluation. Final enrollment in the study was based on written informed owner consent to participation in the study and a final diagnosis of chronic inflammatory enteropathy (CIE) established through the combination of thorough patient history, physical examination, clinicopathologic evaluation (routine blood work, fecal parasitology, and a gastrointestinal panel), diagnostic imaging, and gastrointestinal endoscopy with biopsy collection for histopathologic evaluation. The collection of intestinal mucosal biopsy samples from healthy Beagle dogs (n = 18), serving as a control group, was performed during necropsy after humane euthanasia for unrelated research studies. These clinical trials were reviewed and approved by the Finnish National Board for Animal Experiments under the license numbers ESLH-2007-09833/Ym-23, ESAVI 2010-04178/Ym-23, and ESAVI/7290/04.10.03/2012; tissue samples were surplus materials, and none of the dogs were euthanized for the purpose of this study. The study protocol for including dogs with CIE was reviewed and approved by the same authority under the license numbers ESAVI/6973/04.10.03/2011 and ESAVI/10384/04.10.07/2014.

### Routine diagnostics and sample collection

The CIBDAI score [42] was calculated for dogs presenting with gastrointestinal signs but was not obtained for the healthy controls. This score includes the following criteria: attitude and activity, appetite, vomiting, stool consistency, frequency of defecation, and weight loss, which are graded on a scale from 0 (normal) to 3 (marked changes); the cumulative CIBDAI score can range from 0–18 and is interpreted as clinically insignificant disease (composite score of 0–3), mild disease (composite score of 4–5), moderate disease (composite score of 6–8), or severe disease (composite score of ≥ 9) [42].

Routine hematology (ADVIA 2120i, Siemens Healthineers, Malvern, PA, USA) and serum biochemistry profiles (Konelab PRIME 60i, Thermo Scientific Oy, Vantaa, Finland) were performed in all dogs, and serum albumin concentrations of ≤ 24 g/L were classified as hypoalbuminemia [17]. Endoscopically obtained (CIE group) or necroscopic (healthy controls) gastrointestinal tissue samples were subjected to histopathological evaluation using the World Small Animal Veterinary Association Gastrointestinal Standardization grading scheme [24] and with the pathologist blinded to the patient clinical data. Tissue biopsy samples were evaluated from each gastrointestinal segment that was sampled for morphological lesions and inflammatory changes using a 4-point scale (0 = normal, 1 = mild lesions, 2 = moderate lesions, and 3 = severe lesions). Subsequently, total histological lesion scores were calculated for each gastrointestinal segment and across all evaluated segments.

Clinical outcomes were available for 34 dogs of the CIE group and were used to retrospectively categorize dogs based on the response to treatment as either having food-responsive enteropathy (FRE) if clinically responding to dietary intervention with a therapeutic elimination diet (novel-protein/carbohydrate or hydrolyzed protein diet), antibiotic-responsive enteropathy (ARE) if responding to tylosin treatment (25 mg/kg PO q24h within 3–7 days), immunosuppressant-responsive enteropathy (IRE) if responding to treatment with prednisolone (1 mg/kg PO q12h within 10–14 days) or as non-responsive enteropathy (NRE) if no clinical response to any of these sequential treatment options.

### Measurement of calprotectin in mucosal extracts

Mucosal extracts were prepared as previously described [13]. Briefly, all snap-frozen mucosal samples were homogenized using a Precellys 24 homogenizer (Bertin Technologies, Paris, France) in ice-cold extraction buffer (50 mM Tris/HCl, 150 mM NaCl, 10 mM CaCl_2_, 0.2 mM NaN_3_, and 0.01% (v/v) Triton X-100; pH 7.6) supplemented with a proteinase inhibitor cocktail (1 EDTA-free protease inhibitor cocktail tablet/50 ml of extraction buffer) at a 20:1 ratio (extraction buffer to tissue). After homogenization, samples were centrifuged at 13,000 × g and kept at 4 °C for 10 min; the supernatants were then collected and stored frozen at − 80 °C until analysis of calprotectin using the Bühlmann fCAL® turbo particle-enhanced turbidimetric immunoassay (PETIA; Bühlmann Laboratories, Schönenbuch, Switzerland). The PETIA was performed on a standard clinical chemistry analyzer (Mindray BS-380) with instrument settings as recommended by the reagent manufacturer (Mindray, Nanshan/Shenzhen, China): absorbance measurement at 546 nm and a standard curve based on 6 standard solutions from 0 to 2,000 µg/g. This assay has been validated for use with samples from dogs [43].

The mucosal extracts were centrifuged for 5 min at 3,000 rpm and measured in duplicates without additional dilution. In detail, 10 µL of the extract is mixed with 150 µL assay buffer R1 and pre-incubated at 37 °C for several minutes. A blank measurement was performed after adding 30 µL of the reagent containing the immunoparticles coated with avian polyclonal antibodies against human calprotectin. This measurement is subtracted from the final absorbance measurement after 3.6 min. Extracts measuring above the assay working range (> 2,000 µg/g) were automatically diluted (1:10) by the analyzer and retested.

The total imprecision of the fCAL® turbo PETIA on the Mindray BS-380, as given by the manufacturer, is 6.2% at a calprotectin concentration of 52 µg/g and 1.7% at 418 µg/g. The lower limit of quantification is given as 19 µg/g.

### Statistical analysis

The assumption of normality of data was tested using the Shapiro–Wilk *W* test. Nonparametric analyses were performed because the data was found not to be normally distributed. Differences between mucosal calprotectin concentrations in mucosal extracts from different gastrointestinal segments between dogs with CIE and healthy Beagles were determined by a Mann–Whitney *U* test. A Kruskal–Wallis test or Spearman ρ correlation coefficient was used to test the association of calprotectin concentrations in mucosal extracts with the severity of histologic lesions (graded from 0–3) and clinical outcomes (classification as FRE, ARE, IRE, or NRE) in dogs with CIE. Summary statistics are presented as medians and IQR, or median and range, as appropriate. For all analyses, a *P* < 0.05 was considered significant, and trends were discussed using a *P* < 0.10. A Holm-Bonferroni correction for multiple comparisons or correlations at the same level (*P*_corr_ = *P* ÷ n) was applied if applicable. All statistical analyses were performed using a statistical software package (JMP^®^ v13.0, SAS Institute, Cary, NC, USA; and GraphPad Prism^®^ v9.0, GraphPad Software, San Diego, CA, USA).

## Supplementary Information

Supplementary Material 1.

## Data Availability

All data analyzed or generated during this study are included in this published article and its supplementary files. Original data (anonymized for patient and owner details) are available from the authors upon reasonable request.
