# Supplementary material for: Duodenal and colonic mucosal S100A8/A9 (calprotectin) expression is increased and correlates with the severity of select histologic lesions in dogs with chronic inflammatory enteropathy
Source: BMC Vet Res. 2024 Sep 6;20:393. doi: 10.1186/s12917-024-04256-9 (PMC11378391; doi:10.1186/s12917-024-04256-9)
Supplement: Supplementary file 1 — Supplementary Material 1. [file 12917_2024_4256_MOESM1_ESM.docx]

**Suppl. Table 1 – Correlation of mucosal calprotectin concentrations among the gastrointestinal segments evaluated in the study.** The correlation coefficients (Spearman ρ) for the correlations of calprotectin concentrations measured in mucosal extracts of the different gastrointestinal segments are shown.

|  | **Mucosal calprotectin concentration** | | | | | | |
| --- | --- | --- | --- | --- | --- | --- | --- |
|  | **correlated between** | **Stomach** | **Duodenum** | **Jejunum** | **Ileum** | **Cecum** | **Colon** |
| **Dogs with CIE** | | | | | | | |
| **Stomach** | | – | **0.47** | – | 0.60 | 0.40 | 0.43 |
| **Duodenum** | | **0.47** | – | – | 0.39 | 0.54 | 0.22 |
| **Jejunum** | | – | – | – | – | – | – |
| **Ileum** | | 0.60 | 0.39 | – | – | 0.60 | **0.70** |
| **Cecum** | | 0.40 | 0.54 | – | 0.60 | – | 0.26 |
| **Colon** | | 0.43 | 0.22 | – | **0.70** | 0.26 | – |
| **Healthy control dogs** | | | | | | | |
| **Stomach** | | – | 0.03 | -0.50 | -0.71 | -0.49 | -0.20 |
| **Duodenum** | | 0.03 | – | 0.49 | 0.35 | 0.14 | 0.40 |
| **Jejunum** | | -0.50 | 0.49 | – | **0.78** | -0.30 | 0.35 |
| **Ileum** | | -0.71 | 0.35 | **0.78** | – | 0.03 | **0.48** |
| **Cecum** | | -0.49 | 0.14 | -0.30 | 0.03 | – | 0.66 |
| **Colon** | | -0.20 | 0.40 | 0.35 | **0.48** | 0.66 | – |
| Correlation coefficients highlighted in red indicate a statistically significant correlation (*P_corr_*<0.05), and those coefficients highlighted in green indicate a trend for a significant correlation (*P*<0.05, but *P_corr_*>0.05). | | | | | | | |
